# The roles of health maintenance organizations in the implementation of a social health insurance scheme in Enugu, Southeast Nigeria: a mixed-method investigation

**DOI:** 10.1186/s12939-020-1146-4

**Published:** 2020-03-12

**Authors:** Eric Obikeze, Obinna Onwujekwe

**Affiliations:** 1 Health Policy Research Group, Department of Pharmacology/Therapeutics, College of Medicine, University of Nigeria Enugu Campus, Enugu, Nigeria; 2 Department of Health Administration and Management, Faculty of Health Sciences and Technology, College of Medicine, University of Nigeria, Enugu Campus, Enugu, Nigeria

**Keywords:** Social health insurance, Health maintenance organizations, Southeast Nigeria

## Abstract

**Background:**

In Nigeria, health maintenance organizations (HMOs) are the purchasers of health insurance with a social National Health Insurance Scheme for civil servants. However the roles of HMOs in implementation of social health insurance are not clear. This study determined the roles of HMOs in implementation of the national social health insurance scheme in Enugu, Southeast Nigeria.

**Methods:**

A partially mixed sequential dominant status design was employed in the study. Quantitative data were collected from 613 Federal Government employees that are registered with the National Health Insurance Scheme (NHIS) as part of the Formal Sector Social Health Insurance Program (FSSHIP) using an interviewer-administered questionnaire. Test for sampling adequacy was ensured (KMO, 0.701) and likewise the sphericity of the data using Bartlett’s test (Chi [1] 796.72, *p*-value < 0.001). For the qualitative study, there was document review and in-depth interviews. A total of 28 in-depth interviews were conducted with key stakeholders comprising of managers of HMOs, NHIS manager, providers of health care and personnel in the State Ministry of Health among others. Thematic analysis was used for the qualitative data.

**Results:**

One-third (31.5%) of respondents said that roles of HMOs were very important, while 23% said that their roles were not important. More than half (57.70%) ranked HMOs very low in their roles, while 24.10% ranked them highest. Concentration index shows that the poor were satisfied (−.10), while the rich were highly satisfied (0.13) with roles of HMOs. The qualitative data analysis showed that most of the respondents were not satisfied with the roles of HMOs based on the themes that were developed and analyzed.

**Conclusion:**

There is clear understanding of the functions of HMOs among respondents in the study although they generally think that HMOs are not meeting the expectations of the scheme. There is need for the Federal Government through the National Health Insurance Scheme to provide more effective guidelines for HMOs, supervise and monitor the implementation of such guidelines for HMOs to improve on their roles.

## Background

Health Maintenance Organizations (HMOs) purchase care on behalf of the National Health Insurance of USA [2]. Nigeria adapted the HMO system in 1999. With the formation of the National Health Insurance Scheme (NHIS) [1], private entities were encouraged to form HMOs [3]. The Act establishing the NHIS allowed HMOs to serve as agents to the NHIS and should cover both public and organized private sectors.

HMOs in the Formal Sector Health Insurance Program (FSSHIP) of Nigeria’s NHIS were appointed to give the scheme a private sector face-lift. The founding fathers of the scheme believed that the social system of the country is marred with inadequacies, without checks and balances. Based on that, policy makers in health suggested a system of health insurance with HMOs participating as agents of the NHIS to purchase health services from public and private providers. HMOs are private sector driven and are expected to close leakages that might be arising from poor management by the public sector [4, 5].

The Nigeria health system was initially publicly financed with it attendant problems. The National Health Financing Policy and Strategy of Nigeria recommends that there should be a split between funding and purchasing and the powers for both should not reside in the same agency. It also recommends that there should be a split between purchasing and provision and the powers for these functions should not reside in same agency. Hence, HMOs exist within the insurance scheme to help drive the purchaser-provider split and promote efficiency and sustainability that are not commonly found in the public sector. HMOs purchase care at the primary, secondary and tertiary levels of healthcare. The referral system through the HMOs starts from the primary to secondary and then to the tertiary care. The system is integrated in such a way that NHIS supervises and monitors all the activities that go on in the social health insurance scheme.

There are three levels of HMOs that operate in the country. They are those with National Structure, which are allowed to operate in all the States of the country including the Federal Capital Territory; those that operate within the confines of each of the six geopolitical regions of the country (north central, northeast, northwest, southeast, southwest and south-south); and those who operate within a given State.

Initially, it was envisioned that health insurance would pool funds for the providers who manage patients based on predetermined arrangement, also known as prospective payment system [6]. This method brought about moral hazard, adverse selection, and unwarranted screening of patients in the interest of the providers [7]. To mitigate such inadequacies in health insurance cover, provider-purchaser split was introduced [8, 9]. The purchaser in Nigeria’s health insurance scheme is the HMO. The extent of people’s satisfaction with the roles of HMOs in the scheme is the basis for this study.

HMOs are expected to function based on health insurance principles, which include generation, pooling, purchasing and benefit packaging. *Generation* is carried out through taxes, levies, out-of-pocket payment whereas *pooling* requires organizing the generated funds into a financial basket that gives opportunity to hedge against unexpected healthcare spending [10]. Pooling was also viewed as health system function whereby collected health revenues are transferred to purchasing organizations [11]. *Purchasing* refers to holding the fund and ensuring disbursement to actors in the insurance system [12, 13], whereas *benefit packaging* refers to what should be included or excluded as health services and requirements for referral, co-payment making etc. [14] These are key instruments that steer the health systems towards Universal Health Coverage (UHC) [15, 16].

Purchasing is indeed a function of the HMOs [17]. In Nigeria, HMOs are expected to make a minimum share capital deposit of N100m, N200m, and N400m to function as State, Regional or National HMOs respectively. Whether at the State level or Regional level or National level, HMOs are expected to perform similar roles. The roles of HMOs are defined in the NHIS guidelines [3]. Each HMO is expected to perform within the confines of the guidelines knowing that NHIS has authority to ensure compliance. The questions that come to mind are, do HMOs perform their roles accordingly? Are they acquainted with their responsibilities? Do HMOs aim at ensuring that stakeholders are satisfied with their roles? This study examined stakeholders’ satisfaction with the roles of HMOs in social health insurance implementation in Enugu, Southeast Nigeria. Analysis of roles of HMOs has been done in Nigeria, although the study was mainly a supply-side analysis with interest in processes and roles of actors in implementation of NHIS [18].

However, the extent that different stakeholders are satisfied with the roles of HMOs is not clearly understood. This study examined the roles of HMOs in the implementation of a National Social Health Insurance Scheme in Nigeria. The scheme and HMOs are fully operational in Enugu, southeast Nigeria. Unlike previous studies, the present study incorporated the demand-side by investigating the level of satisfaction with roles of HMOs among beneficiaries and other actors in social health insurance scheme in Enugu State. This study focused on civil servants because they are the only people that are presently fully covered by the Formal Sector Social Health Insurance Program (FSSHIP) of the NHIS. This is because they have income structure that makes deductions for insurance premium easy.

## Methods

### Study design

**Fig. 1:**
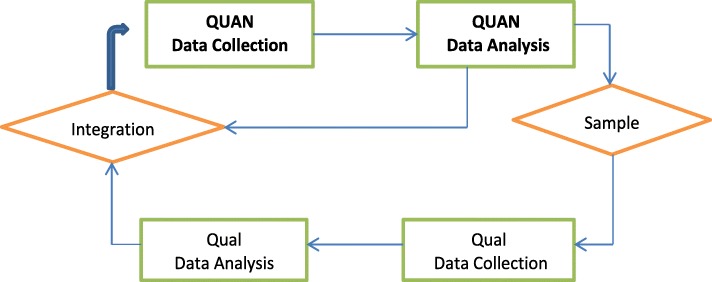
Partially mixed sequential dominant status design (Adapted from Wao & Onwuegbuzie, 2011)

A partially mixed sequential dominant status design was employed in the study [19, 20]. That is, the quantitative phase (surveys) preceded the qualitative phase (interviews) (see Fig. 1). A partially mixed sequential dominant status is a method of research in which both qualitative and quantitative methods are used, but the one that draws more information is said to be dominant. Hence, the dominant phase in this study was the quantitative phase because it had more variables of interest that were generated and analyzed. A pre-tested interviewer-administered questionnaire was used to collect quantitative data whereas in-depth interviews (IDIs) were used for qualitative data collection.

Respondents were asked about their perceptions, roles of HMOs and satisfaction with HMOs in the scheme. The data provided empirical basis for understanding the roles of HMOs followed by thematic analysis in the qualitative phase. Qualitative data were collected through review of literature and IDIs to examine the roles of HMOs from stakeholders’ points of view. Quantitative data were collected from Federal Government employees that are registered with the NHIS. The use of mixed methods approach ensured validity of the findings through triangulation [21]. QUAN in the Figure below represents quantitative method, while Qual represents the qualitative data collection method. The study was conducted in Enugu urban.

### Study area and population

The study was undertaken in Enugu state, southeast Nigeria. Enugu State with a population of 4,1 million [22], has Enugu metropolis as its capital city. The state covers an area of approximately 12,727 km^2^ and is surrounded by six States. It shares a border with Abia and Imo States in the South. It is flanked to the east and west by Ebonyi and Anambra States respectively and in the north by Kogi and Benue States. More than 90% of the inhabitants are Christians, while Muslims and Traditionalist share the remaining 10%. The native population is entirely Igbo with a sprinkling of Igala near the borders with Kogi State. Other ethnic groups are however well represented in the State with a predominance of Hausa and Yoruba communities. The State is well known for its industrial centres and markets with 75% of the State being agrarian.

The study population was the Federal civil servants in Enugu metropolis, and they were approximately 18,000 in number [13]. Enugu metropolis is predominantly populated by civil servants that are employed by the Federal, State and Local Government Areas (LGAs). The survey tool was administered to Federal Government employees enrolled in the NHIS that reside in the Enugu metropolis. Federal Government staff, which by virtue of their job are enrolled with the NHIS, were approached and interviewed. Interviewees in the quantitative phase included staff of University of Nigeria Teaching Hospital, National Orthopaedic Hospital, Federal Ministries (agriculture, education, finance, etc) staff, Police, Army, and Federal Road Safety among others.

Information on functions, objectives and effectiveness of HMOs, were elicited through IDIs with key stakeholders including NHIS managers, CBHI managers, HMO manager, health care providers in selected hospitals and personnel in the State Ministry of Health. The design of the FSSHIP of the NHIS also allows the private sectors to enroll and make use of the accredited HMOs if they wish to. Communities or organizations that develop community-based health insurance schemes (CBHI) can also make use of HMOs if they want. Hence, the central point of this study is investigating the roles of HMOs in the implementation of the scheme. The sample was selected from a representative sample of the people that are in the formal sector.

### Sampling for quantitative survey

Enugu State has a population of approximately18000 Federal Government employees who are expectedly covered by NHIS. A sample size of 581 employees was used for the study; determined using Cochran formula corrected for finite population [23]. For the computation, z = 1.96, *p* = 0.5, ɛ = 0.04.

$$ {n}_0=\frac{Z^2p\left(1-p\right)}{\varepsilon^2}\sim Cochran\ for mula\ for\ sample\ proportion\ for\ large\ population $$

$$ n=\frac{n_0}{1+\frac{n_0-1}{N}}\sim the\ for mula\ for\ correction\ for\ finite\ population $$

$$ \upvarepsilon =\mathrm{desired}\ \mathrm{level}\ \mathrm{of}\ \mathrm{precision}\ \mathrm{p}=\mathrm{estimated}\ \mathrm{proportion}\ \mathrm{of}\ \mathrm{the}\ \mathrm{attribute}\ \mathrm{in}\ \mathrm{the}\ \mathrm{population} $$

### Selection of participants for quantitative survey

The sample of beneficiaries was selected using a 2-stage process. First, employers were selected with probability proportionate to size (number of employees). Then, a random sample of employees was selected for the beneficiary survey. Data that were provided by NHIS expectedly indicated the total number of Federal employers in Enugu State.

The initial sampling frame was made up of a list of NHIS beneficiaries by employer, in Enugu state. The list was provided by Zonal office of the NHIS that is located in Enugu metropolis. We assumed that an “employer” is in a single location with the following exceptions:

- Nigerian Police Force has employees which are posted to police stations throughout the State. A distinct work location (e.g. police station or HQ) was a cluster
- Hospitals – these are also large employers, so we took the department as a cluster (allowing > 1 cluster for a facility)

The number of clusters was determined by feasibility. We thought it was logistically possible to survey greater than 12 clusters in total. Each employer in the NHIS list was treated as a cluster with the exception of the police, where each police station (and the central authority) was treated as a cluster.

The sample was stratified to ensure that the largest employers (e.g. police and hospitals) were selected. There were 4 strata altogether:

- Police (with each police station being a cluster)
- Army
- Hospitals (Psychiatric hospital, Orthopaedic hospital; University of Nigeria Teaching hospital)
- All other (the assumption being that these are mostly administrative offices or teaching institutions) complemented.

Clusters were selected with probability proportionate to size (number of employees), and within a cluster a fixed number of people were sampled. This gave a self-weighting sample (small employers have a lower probability of being sampled, but within that unit, each individual had a larger probability, and these cancel each other out to allow an unweighted analysis).

Because the marginal cost of sampling additional individuals was very low once an institution had agreed to be included in the survey, we proposed to target to survey 30 clusters X 20 people each (600 in total). Because there were some small employers with fewer than 20 people, this excess sampling allowed us to get closer to the target sample size of 600. The number of clusters per stratum was selected by identifying the number of workers, percentage of workers and number of clusters selected.

The procedure for selecting clusters was by constructing a list of all the clusters with the number of employees in each. This required updating the list from NHIS, and also visiting the large employers (Army, Police, and Hospitals) and getting the breakdown by location (Army and Police) and department (hospitals).

Once a cluster was visited, the aim was to select 20 employees. If there were fewer than 20 employed, everybody qualified to be interviewed. If they were more than 20, we selected 20 using systematic random sampling. To do this:

- We made a listing of the employees in the firm (cluster)
- Calculated the total number of employees/20 (this is the interval)
- We generated a random number between 0 and the interval (= x)
- The first selected employee was the number x on the list, the second is the number (x + interval), the third is (x + 2*interval), etc.

For example:

If the number of employees = 220 then the interval = 220/20 = 11. Choosing a random number between 0 and 11 (e.g. 5), then the first employee would be number 5; the second 16 (=5 + 11); the third 27 (= 5 + 2*11); the fourth 38 (= 5 + 3*11), and so forth.

The study examined the level of satisfaction with HMOs among the beneficiaries who are Federal staff. The regression model was thus given as:

Logit (satisfaction with HMO) = b_0_ + b_1_(gender) + b_2_(household size) + b_3_(age) + b_4_(education) + b_5_(occupation) + b_6_(premium) + b_7_(satisfaction with provider) + b_8_(no. of other members covered) + b_9_(any other members covered by federal health insurance) + b_10_(any other insurance cover) + b_11_(heard about HMOs) + b_12_(socioeconomic status) + error.

Socio-economic index was determined using Principal Component Analysis (PCA) to ascertain equity in health care delivery among the enrolees. The level of equity was determined using Concentration Index (CI). Concentration Index measures the level distribution of the variables of interest across the socioeconomic groups. CI with a positive value indicates that variable of interest is distributed in favour of the rich. If calculated CI has negative value, it means that variable of interest is in favour of the poor.

Test for sampling adequacy was ensured (KMO, 0.701) and likewise the sphericity of the data using Bartlett’s test (Chi^2^ 796.72, *p*-value < 0.001). This was done using variables on asset holding, consumption pattern and income of respondents. SPSS (version 21) was used to enter the data and subsequently transferred to Stata (version 12) software for analysis.

### Sampling for qualitative survey

#### Document review guide

We reviewed policy documents such as NHIS Guidelines, HMO Operational Manual, Enugu State PPP Policy, Federal and State Health Financing and Equity Policies and National Health Act were reviewed and they described the operation of the NHIS and HMOs. This enabled the authors to understand procedures of the scheme.

#### In-depth interviews (IDI)

IDIs were conducted with the key actors in the scheme. Those included in the interviews were key decision makers at the State NHIS; HMO managers that registered providers in Enugu or their representative; directors of a sample of provider facilities or their representatives in Enugu. We listed all the HMOs and provider facilities that are registered with the NHIS in Enugu State. Because there are a good number of providers, those who were randomly selected but could not be reached were systematically replaced. HMO managers in the State (8 of them) and 15 healthcare providers were approached and interviewed. The State has Community Based Health Insurance (CBHI) as well as Faith Based CBHI. The managers of the CBHI were also included in the interview as they were expected to provide valuable information about the roles of HMO. We included respondents in CBHI and Faith Based CBHI because the guidelines for HMOs operation allow them to purchase care for such private establishments. We interviewed the managers of CBHI and Faith Based CBHI to also determine their position with respect to roles of HMOs that may have engaged with them.

The main objective of these interviews was to determine roles, objectives and extent of involvement of HMOs in the scheme. What made the respondents feel satisfied or not satisfied with HMOs were determined through the interviews. Respondents who were HMO representatives were coded as HMO1, HMO2 etc. Respondents who were health care providers were coded P1, P2 etc.; CBHI managers who participated were referred to as CB1, CB2 etc.; NHIS managers interviewed were referred to as NH1 and NH2 while the personnel in the Ministry of health was referred to as HI (Table 1).

**Table 1:** Summary of Data Collection Methods per Specific Objective

Objective	Data required	Design	Population	Sample size	Sampling Approach	Tool	Analysis
To determine levels of satisfaction on HMOs by different socio-economic groups in the State	Available information and communication across socio-economic groups that are covered, satisfaction with roles of HMOs	Quantitative (using SPSS and Stata)	Survey of Federal Staff who are registered with NHIS using the enrollee register from the NHIS	Survey with enrollees of Federal Staff for NHIS (613 respondents)	Purposive (multi- staged sampling techniques)	Questionnaire, pen, Stata, SPSS statistical packages	SPSS, Stata
To determine roles and responsibilities of HMOs in Enugu State	Roles responsibilities and institutional requirements	Qualitative (IDIs using N-vivo)	Stakeholders in social and private Health Insurance including HMOs, providers, CBHI trustees and NHIS managers.	28 in-depth interviews involving stakeholders	Purposive	IDI guide, tape recorder, pen,	N-vivo
To determine the extent of HMOs’ functions in implementation of social health insurance schemes	Levels of involvement/participation in the scheme by HMOs and other stakeholders	Qualitative (IDIs using N-vivo)	Public and private health care providers in the study area; mutual health organizations and key opinion leaders	Same as above	Purposive	IDI guide, tape recorder, pen	N-vivo

## Results

### Quantitative findings

#### Socioeconomic characteristics of respondents

All respondents had formal education and 38% were females. Average number of people in household was 4.39. Average age of respondents was 39.53 years. The respondents were divided into quartiles representing their socioeconomic groups (as seen in Table 2).

**Table 2:** Socio-economic and demographic characteristics of respondents

Variables	*n* = 613	%
Attended School	613	100
Female respondent	231	38
Main occupation		
Cleaner	8	1.30
Clerical	70	11.40
Middle manager	53	8.60
Professional	182	29.70
Senior manager	30	4.90
Other	270	44.00
Other source of income		
Farming	27	48.20
Petty trading	17	30.40
Business	9	16.10
Part-time job	1	1.80
Others	2	3.60
SES distribution		
Q1 (poorest)	156	25.45
Q2 (very poor)	152	24.80
Q3 (poor)	156	25.45
Q4 (least poor)	149	24.31
No of people in Household Mean (SD)	4.39 (1.96)	
Age: Mean (SD)	39.53 (8.29)	
Take home salary: Mean (SD)	78,255.90 (58,682.57)	
Extra income of respondent Mean (SD)	5052.32 (14,629.08)	
Number of household members covered: Mean (SD)	2.78 (1.77)	

*Note: many of the respondents in the selected clusters (in* Table 2*) did not fall into the category of respondents in the study instrument. For instance, in the military, police and Federal Ministries, some cadres such as officer cadet, sergeant at arm, mortuary attendance etc, were not included and so were referred to as others*

In Table 3, more than half (53.0%) rated the roles of HMOs as important. Almost one-third of the respondents (31.3%) indicated that roles of HMOs were very important whereas 23.0% viewed HMOs’ roles as not necessary. In terms of ranking the level of roles of HMOs from 1 to 5 (where 1 represents 20%, lowest and 5 represents 100%, highest), more than half (57.70%) ranked HMOs very low, while 24.10% ranked them highest.

**Table 3:** Satisfaction with roles and ranking of HMOs by respondents

	Respondent	%
Roles of HMOs		
Very important	192	31.3
Important	157	25.6
Not necessary	141	23.0
Not needed	104	17.0
Can’t say	19	3.1
Chi2 (*p*-value)	44.09 (0.000)	
Ranking of HMOs		
Lowest (20%)	108	17.6
Very low (40%)	329	57.7
High (60%)	18	2.9
Very high (80%)	10	1.6
Highest (100%)	148	24.1
Chi2 (*p*-value)	221.51 (0.000)	

Level of satisfaction is not statistically significant among those who are highly satisfied (*p* = 0.64). Concentration index shows that the poor are satisfied (−.10), while the rich are highly satisfied (0.13). These can be seen in Table 4.

**Table 4:** Satisfaction with HMO by different Socio-economic groups

Quartile	Highly satisfied N (%)	Very Satisfied N (%)	Satisfied N (%)	Not satisfied N (%)	Highly not satisfied N (%)
Q 1 (poorest)	4 (21)	19 (18)	46 (33)	41 (26)	46 (24)
Q 2 (very poor)	4 (21)	28 (27)	39 (28)	50 (32)	31 (16)
Q 3 (poor)	4 (21)	44 (42)	24 (17)	37 (24)	47 (25)
Q 4 (least poor)	7 (37)	13 (13)	32 (23)	29 (18)	68 (35)
Total	19 (100)	104 (100)	141 (100)	157 (100)	192 (100)
X2 (*p*-value)	1.68 (0.64)	23.94 (0.00)	9.62 (0.02)	7.52 (0.06)	22.98 (0.00)
Concentration Index	0.13	0.01	−0.1	− 0.07	0.11

Table 5 shows logistic regression model of level of satisfaction of the respondents with the roles of HMOs. The overall estimate is statistically significant showing Chi,^2^ prob. >Chi^2^ and Pseudo R^2^ values of 163.86, 0.000 and 0.231 respectively. The Table also shows that education, occupation and socioeconomic status of respondents were statistically significant at 95% confidence interval.

**Table 5:** Logistic regression model of satisfaction with roles of HMOs

Satisfaction with HMOs	Coefficient	Std. Err.	z	P > ızı	95% confidence interval	
Gender	− 0.033	0.232	− 0.14	0.886	− 0.489	0.423
No of people in houshold	−0.030	0.071	−0.43	0.670	−0.168	0.108
Age	0.023	0.014	1.59	0.112	−0.005	0.051
Education	0.477	0.087	5.50	0.000	0.307	0.647
Occupation	0.166	0.082	2.02	0.044	0.004	0.327
Premium	0.086	0.443	0.19	0.846	−0.782	0.954
satisfaction with providers	−0.342	0.117	−2.91	0.004	−0.571	−0.112
number of others covered in hh	−0.321	0.078	−4.12	0.000	−0.474	−0.168
any other insurance members	−0.728	1.084	−0.67	0.502	−2.854	1.398
heard about HMOs	1.672	0.220	7.59	0.000	1.24	2.103
socioeconomic status	−0.209	0.106	−1.97	0.048	−0.416	−0.002
Constant	−2.069	1.030	−2.01	0.045	−4.088	−0.489

No of obs. = 582; LR Chi2 = 163.86; prob. > Chi2 = 0.000; Pseudo R2 = 0.2

### Qualitative data analysis

In the qualitative phase, thematic content analysis was employed to examine the functions of HMOs, achievement of HMOs objectives, effectiveness of HMOs, and rating of HMOs. Table 6 presents a breakdown of participants interviewed.

**Table 6:** Summary of those involved in the interviews

S/No.	Participant	Short form	Number interviewed
1	HMO	HMO	8
2	CBHI representative	CB	4
3	Health care provider	P	13
4	NHIS Managers	NH	2
5	Ministry of Health personnel	HI	1

#### Functions

HMO’s are to ensure accreditation of providers as well as visit them to make sure that the environments are within the NHIS guidelines. Their other functions include fishing out irregularities in the system and reporting same to the NHIS for enforcement and discipline. HMOs are to allocate funds to players in the scheme particularly the health care providers. They are meant to provide access to health which requires having doctors and other personnel available in health facilities. Although HMOs have specific functions as mentioned above, such functions are noted to be deficient in performance. CB1 (Community informant number 1) for instance said that to a great extent,

*“HMO that the community signed up with was not performing its functions in the community insurance scheme”.* Further to that he said: *For their services to be retained, they would definitely have to do more. If you don’t press for them to look in and see what the doctor is doing, or what is going on there, they will not care".*

With respect to their functions and getting things done properly and providing required monitoring and inspection of the facility, CB1 said they were not doing well. According to him, *“they are to some extent getting better as PATHS2 from Enugu Ministry of Health came and again reminded the HMO of their responsibilities and why they should stick to that for the system to function effectively”.*

Respondents said that HMOs in their functions should take care of enrollees based on the regulations, and that the system should be such that enrollees are not allowed to change their primary healthcare more than twice a year as HMOs should keep close monitoring. But does that really happen? One would ask. According to P3 (provider number 3), “*some of those who come here are not even properly guided by the HMOs on what the guidelines of the NHIS said with respect to their rights and privileges and when they could change provider and for what reason?”*

Their other function includes fishing out irregularities and reporting such to NHIS for enforcement. Again it was observed that HMOs perform the function of purchasing and allocating of funds to the players in the scheme as well as conducting quality assurance and sanctioning of providers that default. They are also meant to conduct interactive forum with the enrollees and request them to provide the list of defaulting health providers. In line with that, HMO1 (HMO manager number 1) said their HMO *“takes care of the enrollees based on the regulation - the primary provider refers to the secondary provider who in turn refers to tertiary provider”.*

Dissatisfaction was reported about the functions of HMOs because of non-compliance with NHIS guidelines. HMO3 gave an instance of what happened as they went for their statutory inspection of the accredited hospital and discovered that *“a certain hospital was made of mud blocks and was a personal house with family members. That kind of ‘hospital’ has life and they receive capitation against the rules of NHIS”.*

A major function of HMOs according to HMO5 is allocation of funds to the players in the scheme. Without HMOs, such function would not be effectively performed. According to him,

*We have schedule of how the money is being divided. Part of that money goes for the capitation, which goes to the health care provider. Part of it is for fee for service which is being used, if the primary health care didn’t do anything, it goes to the secondary health care which is for the services provided. Part of the money in case of CBHI, goes for the BoT which is about 10% of the money they paid while a part goes for the admin fee, which is for HMO. That is how this capitation is done. 60% goes for capitation, 20% goes for fee for service, which means the bulk money they contribute, 80% is for health care services.*

HMO7 believes that in addition, they conduct quality assurance, and that *“if providers are found wanting in any area, they would be corrected. Our functions also include education of providers, and if they are found wanting in any way, they are given proper directives”.* She also said that they go out to have interactive forum with the enrollees, who make their complaints, and more often they request them to mention the hospitals involved and they try to sort out whatever issues they may have.

P8 said that in terms of functions of HMOs, it includes coming to the facility for inspection, which happens in some cases. According to him, *“They come and look and find those you have attended to and how many enrollees”.* Further to that, P8 said that:

*HMOs are wired to make money out of the system. (To him), their job is not to see to patient and what should be done to save the patient. Their interest is only on how they can make money and that is what our people do not understand. That’s their own. It is those that are health workers (that is ogbuebule), their job is to attend to the sick. They take care of the sick that is what they do. That of HMOs is how to make money. But people don’t understand. So you should not put such people to be taking care of the sick. It won’t work. They only want money".*

The investigation shows the level of understanding of the functions of HMOs among the respondents. They generally demonstrated good knowledge about the functions of HMOs. In some cases they believe that HMOs’ roles are clearly stated by the guidelines of the NHIS even though they do not believe that HMOs are living the expectation of the stakeholders. It is however important to know that good knowledge about the functions of HMOs would help in determining how much they have been able to achieve their objectives. This is because examining how far they have been able to achieve their objectives will also show that they are either performing their functions or not. It was also necessary to buttress the arguments on the functions of HMOs. The study also looked at how much HMOs have been able to achieve their objectives.

#### Achievements of HMOs’ objectives

The NHIS guidelines spelt out what every stakeholder should do. By that, it is objective of the HMOs to ensure that providers do not complain about remittance of their capitations or fee for service. Their objectives also include wider coverage and ensuring universal health coverage and making sure that enrollees do not complain about quality of service and out-of-stock syndrome.

To achieve the set objectives, there should be some form of monitoring to ensure that policy guidelines are followed by the stakeholders particularly the HMOs who are referred to as purchasers. Negative judgment about the roles of HMOs indicates that objectives are not really achieved. Inability to achieve objectives might stem from disregard to existing contracts between stakeholders which in most cases is not acceptable. It might also be that flow of funds as agreed by the parties is not happening. Feelings of the beneficiaries and the way the entire system functions could also determine the level to which objectives are being achieved. Again number of enrollees at any point in time is a measure of achievement.

Extent of achievements of objectives of HMOs and NHIS was examined with many saying that little have been achieved. With respect to remittance of capitation as agreed with NHIS, HMOs are not performing as required, which means their performance is below expectation.

Respondents expressed concern about the flaws of HMOs in achieving their objectives. One of them was systemic failure of the scheme occasioned by inadequacies of HMOs. Achieving the objectives as it were can only happen if there are laid down principles that must be followed. P5 suggested that this happens because *“NHIS and HMOs and even the providers are not investigated and that is why they do not even aim at achieving any objectives”.*

Notwithstanding, some others like P13 said that the system is not completely a flop as assumed. He maintained that *“even though the scheme is not currently efficient, things will still change with time”*. To a large extent they agreed that HMOs are achieving their objectives in that they are promoting access to health seeking, and P1 maintained that, *“If you are insured, you are better willing to go for services than when you are not insured, in the sense that you don’t have to be paying this one or that one. I think the NHIS will go a long way in helping the health sector in Nigeria”.* Others also maintained that HMOs are only trying to achieve their objectives. In the words of P2, *“HMOs on their own are trying but then I know that some HMOs are becoming deficient in terms of the payment. At times they owe. They owe. So for me they are derailing from what they agreed ab initio”.*

Even HMOs themselves believe they are not completely complying with the set objectives. HMO2 said

*In as much as HMOs are doing work, even HMOs on their own, sometimes are not even meeting up in that regard - maintaining quality assessment and ensuring quality assurance. Of course there are a lot of complaints coming from enrollees about the scheme’s non provision of drugs, out of stock syndrome and being segregated from normal patients in some cases which is actually not meant to be.*

This statement shows that objectives of HMOs are not achieved from many points of view. This again was agreed to be happening because NHIS is not strong in its supervisory roles which includes ensuring that HMOs are compliant with the set guidelines.

P5 believes that flaws in the objectives of HMOs are systemic and has much to do with Nigerian factor. According to him,

*“We used to say health for all by year 2000. So when we got to year 2000, we shifted it. So when we got to 2000 we shifted it to 2015 with MDG. They knew that Nigeria is not working. When we got to 2015, they said, let us go to 2020. All government health facilities are not working and nobody cares. You can only achieve an objective if there are laid down principles that must also be followed”.*

He believes that NHIS and HMOs and even the providers are not investigated and that is why they do not even aim at achieving any objectives.

However, P13 believes in the system and said that even though it is not currently efficient, things will still change with time. He said that

*“it is a new idea in Nigeria, so it will take a lot of time to gradually work the way all the people in the relationship - NHIS, HMOs and health care providers would like. Once everybody understands the roles properly, it will be a seamless thing”.*

This assertion however shows some level of negative judgment about HMOs with reference to achieving their objectives at the moment. Lending voice to that, P8 asserted that he was totally uncomfortable with the system and the roles of HMOs. He maintained that:

*They are not doing it well because there are some unacceptable things they do. They are not doing it well. Like they don’t meet up with the contractual arrangements they have with providers. At times those enrolled in your hospital, they will not give you all of them. They remove some of them. I am not sure they still do that. So that they will not pay you all they are supposed. What they do in short, is like somebody in the market who says this is the quantity I will buy and this is the amount you will pay. So you tell doctor this is what you will be charging for this or that, whereas you are not a doctor. That is the kind of thing they do and other things.*

Actually achieving objectives includes getting wider coverage or having universal coverage. But from the look of things and the way the respondents feel, much has not been done to achieve the desired objectives. P11 said that a lot still needs to be done to achieve the objectives. In his words,

*They have not done much. There is a lot more to do. This is because NHIS covers mainly federal civil servants. The state civil servants and LGAs workers are not included. Instead, there is a room for voluntary enrollees but the awareness is too poor, too poor. Vast majority of Nigerians do not have access to these insurance, and they are the poorer ones. In fact those who don’t have access to it are even the poorer ones, those who don’t have money. The middle class and I mean the federal civil servants are those who are opportune to have access to social health insurance. Another small number, corporate organizations have access, but it is still small compare to the population of the country. So by and large, we haven’t done enough, a lot more to be done in health care provision****.***

Notwithstanding, there are respondents who believe that HMOs are meeting their objectives. Such proponents believe that HMOs are helping to provide access to health care for more people. HMO5 said that in the aspect of CBHI, where his HMO works, people are having more access to health care than what used to be. He said,

*“A lot of people are keying in, and we are able to solve the medical needs of so many people. When we started at the community, I think we started with 18 people, but many people are coming in. Many people are getting aware of it, and they are coming in. We keep on increasing, up to 300, 400 a month”.*

P9 also believes that the scheme is working and that the HMOs are trying their best. He believes that getting to the desired level in terms of objectives of HMOs will happen in a matter of time. P10 also believes that HMOs are meeting their objectives. He said that they meet “*… .because they pay their capitation as at when due. I receive my alert every month, just that a number of enrollees sweep the amounts. The beneficiaries come for treatment. But the problem is that the percentage of Nigerians covered is still very small”.*

There is a level of acceptance of HMOs with regards to what they have been able to achieve, but generally, respondents think that much still need to be done. For instance, in the area of coverage, which is part of what is stated in the guidelines of NHIS, little or nothing has been done. It is believed that those who actually need health insurance because they are vulnerable are yet to be covered. The NHIS has it in the guidelines that the scheme should spread and get down to every level of the economy. However, this can only be achieved by HMOs meeting up with their objectives. What constitutes effectiveness was also examined by the respondents. To a large extent understanding effectiveness of HMOs will help explain how far they have been able to achieve both their functions and objectives.

#### Effectiveness of HMOs

Effectiveness demands that those who are qualified and are experts are given opportunity to perform the required function. Being compliant is a good determinant of effectiveness in any system. This means that any form of default due to inadequacies of the system would not be acceptable. Effectiveness requires up-to-date information about how a programme functions and if possible a data base from where information could flow. In health insurance, part of effectiveness lies in level of pooling. Where a system is seriously fragmented like in the case of Nigeria’s health insurance, effectiveness may not be achieved. It is expected that a synergy should exist between all the health insurance components (CBHI, NHIS, Faith-based and private sector) for a more organized and effective system. Being able to settle bills is an indication of effectiveness in insurance scheme. The extent to which people have been organized to participate in health insurance is also a measure of effectiveness. Methods of reimbursement that does not amount to unnecessary delays can be adjourned to be part of effectiveness.

There are views indicating that HMOs have not been effective. The following lines of thought were expressed by CB1 about HMO. *“It is always good that expert is there, but let them do the work to justify the pay. I don’t want to take someone else’s job, otherwise if somebody will not put in to justify what is being paid for, then some other ways may be developed”.*

In another line of thought, HMO1 believes there is inefficiency in the way HMOs conducts the process of health insurance. In his words,

*NHIS is doing great. But HMOs are not doing well also. The hospitals should have their data on computer and HMO can see it online. If possible, use your thumb print and use cards on the web so that everyone will know that is indeed malaria. How do you confirm a patient without computer? There is need for monitoring and evaluation.*

On the other hand, HMO4 believes HMOs are performing and are effective. According to him,

*“Well, without HMOs, the scheme would have been moribund like every other government project. Some people woke up to stark revelation that if HMOs had not been in this business, that it would have been like every other government project. It is sustainable now because of the private sector - HMO”.*

Their effectiveness should actually be measured by the extent they have been able to bring people to access health care. P7 believes the HMOs and the scheme generally have made health seeking more effective. And he said, *“I think they have done quite a lot because it has made many people to have access to medical care. Many more people that could not, now have access to medical care”.* The system of payment in the scheme by HMOs was however considered as ineffective. P8 said he does not have people on admission but those who do are not finding it easy with the HMOs due to ineffectiveness in payment. He said**,***“my colleagues who give admission say that they usually encounter some problems. They say that they are not paid regularly, and that they even cut the bill. That is where the problem lies”.*

#### Rating of HMOs in a scale of 1–5

Apart from examining the effectiveness of HMOs, the study also rated HMOs on a scale of 1 to 5. Rating here has to do with utility attached to roles of HMOs and how much they have been able to perform their roles in the system. The highest rate was 5 while the lowest rate was 1.

Respondents rated HMOs’ roles differently. P2 rated HMOs 3 on the 5-point scale. P5 rated them 50% and said their low performance was because of the Nigerian factor where anything federal does not work. In his words, *“HMOs are now the people who carry the load of NHIS”.* This implies that to a great extent, NHIS does not do much that is expected from it and has therefore created room for HMOs to be performing more responsibilities than expected.

P6 rated the HMOs 60% because he feels much still needs to be done not just by HMOs but also by the enrollees, providers and NHIS. NH2 scored HMOs 75% while HMO5 scored self- 80%. P8, on the other hand, scored them average and said, *“Well I will not pull them completely, because there are things they do that are good. I will not score them more than 50 percent. I could have given them less than 50”.*

HMO7 rated HMOs 95% and said that remaining 5% is because they are not yet perfect. She maintained that the level of success in the scheme was because HMOs give the insurance system some private touch. The rating however indicated the feeling of actors in the scheme and how they perceive HMOs and their roles. It is important, however, to know that the responses are diverse feeling of the respondents, which in turn were based on their individual experiences.

## Discussion

The study investigated the roles of HMOs in the implementation of social health insurance scheme in southeast Nigeria. It has been noted that the reason for establishment of health insurance is to make health care more accessible, affordable and equitable across socioeconomic groups [24]. However getting this achieved in the low and middle income countries requires involving the private sector. Our study examined the roles HMOs play as private sector in NHIS implementation in Enugu State. This was done by examining stakeholders’ satisfaction with the roles of HMOs; rating of their roles as well as examining functions, objectives and effectiveness of HMOs.

The quantitative study revealed the roles of HMOs based on the level of satisfaction that respondents have. This was done by cross tabulation of the satisfaction with variables of interest. The outcome of the investigation showed that respondents were not generally satisfied with the roles of HMOs and they have their reasons. There is therefore a need to address issues that make respondents not be satisfied with roles HMOs so as to improve on the overall objectives of social health insurance.

In equity driven investigation, it is a major concern to determine the extent to which the poor are affected by any health care decision. In this study, there is evidence that the poor are not satisfied with the roles of HMOs. Further analysis to determine the level of satisfaction with HMOs based on the responses of different socioeconomic quartiles showed that respondents were not actually satisfied with the roles of HMOs. This could be attributable to their level of perception about the roles HMOs on the one hand and their encounter with the scheme, which probably were unacceptable on the other hand. Logistic regression model also confirmed the level of satisfaction with HMOs, indicating that education, occupation, number of people covered in a household and socioeconomic status of respondents played significant roles in determining the level of satisfaction with the roles of HMOs in the scheme. This shows that how much people are satisfied with HMOs has to do with their socioeconomic characteristics including educational attainment. By extension, if beneficiaries are satisfied with the scheme, more of them will be using the services of the accredited providers. It is expected that increasing the level of satisfaction will therefore promote access to health care and ensure UHC [25]. Currently there is evidence that the poor in sub-Saharan Africa bear the highest burden of diseases and experience high level of catastrophic health expenditure [25]. Social health insurance has been considered a major option for achieving UHC through financial risk protection. This demands that risk protection should not leave doubts on the extent of benefit by the people especially the poor and vulnerable [26].

Satisfaction with roles and ranking of HMOs were to show the level of feeling about the roles of HMOs. People expressed dissatisfaction with the scheme generally. There is apathy about the roles of HMOs. This probably informed the rating of performance over the years. For instance, it is expected that HMOs should provide some level of efficiency through monitoring of activities of providers and checking unnecessary use of health care services or moral hazard on part of beneficiaries [7]. This, to a great extent has not really happened and has actually resulted to low quality and low performance of the accredited providers and the system in general. Even across the socioeconomic groups, overall level of satisfaction of the respondents was not encouraging. This can also stem from the level of support that the scheme receives from the public sector. Study across sub-Saharan Africa shows high level of private health expenditure in health with Nigeria (78.3%) being on top of other countries of Ghana, Kenya, Tanzania and Uganda [27]. This indicates that even those that are covered by any form of health insurance still make substantial out of pocket expenditure. Satisfaction with HMOs could also include the ease at which the respondents are able to access health care without out paying out of pocket. Decisions that aim at improving health seeking by the people will go a long way in promoting access to health care across socioeconomic groups.

In-depth interviews showed that HMOs are trying to justify their roles and they believe that they are performing in their responsibilities. This line of thought was mainly expressed by HMOs that participated in the study, and would not be taken as overarching reasons especially when compared to other responses. It was also expressed in the qualitative analysis where they doubted if they have been able to achieve their functions. There was clear understanding of what should make up their functions, but the respondents do not really agree that the functions are being performed within the confines of the scheme. Their view can be aligned with existing empirics that see Nigeria health system as performing below the standard [28], which indicate that quality and efficiency in service are not assured, and this has continued even with the introduction of HMOs in the health financing mechanisms.

The primary objective of HMOs in line with the NHIS guidelines is to ensure access and quality healthcare irrespective of peoples’ socioeconomic status [3]. The extent they have been able to achieve the objectives is not very encouraging. Many of the respondents see the HMOs as thinking of their pecuniary interest than providing the required services for setting them up. They consider the HMOs as being highly profit oriented which has made it very difficult for them to render good services without compromising quality [29]. Their activities in some cases do not give them the required stand to demand for quality and efficiency from the providers.

Rating HMOs among the respondents both in the quantitative and qualitative phases did not show strong acceptance of their roles. Most of the quantitative respondent rated them very low. This was further affirmed by those in the qualitative study. Generally, they believe that HMOs have not scored up to average, even though none would score them zero. The reason for the low scores stems from their level of perception of the roles of HMOs and how much they have been able to achieve in the scheme implementation. Overall it is expected that they should be up-and-doing especially in the areas of monitoring of the providers and quality of services that are provided to the enrollees.

These however do not mean that HMOs have failed completely. In some cases, the respondents, even provider confessed that HMOs’ presence in the scheme was a major reason why the scheme has not flopped. Their general assumption is that government establishments do not last for long unless there is public private partnership. Some of the respondents believe that HMOs have actually provided the needed private sector initiatives, but would need to do more especially when it comes to efficiency and effectiveness of the system. These do not negate the need for them to improve on their roles in the scheme for a better health insurance system across the country. Improvement on the roles of HMOs could also be made if NHIS could attach some level of performance based financing to the responsibilities of HMOs. This system has showed that compliance with the rules of engagement could promote efficiency and UHC [30].

This study showed some level of contrast about how HMOs function in both quantitative and qualitative components. In the quantitative component of the study, the respondents were beneficiaries who believe generally that the system is not satisfactory. The reason for their position stems on their interface with providers and not necessarily the HMOs. Analysis in terms of knowledge about the roles of HMOs showed the beneficiaries have limited knowledge about what HMOs do. That does not however negate the point that their level of satisfaction cannot be determined only by what they gain from the HMOs directly but rather by what they gain from the entire system.

On the part of respondents in the qualitative component, they believe that HMOs have not completely failed but need to be encouraged to do better. This argument was posited both by the HMOs themselves, NHIS managers and some of the providers interviewed. Far reaching consensus among the qualitative respondents was that existence of HMOs has put private touch to the insurance system. Their response to a great extent supports the clamour for public private partnership that would make the health system more effective and efficient. They believe that HMOs involvement is a move in the right direction but that NHIS should step up their management processes particularly in the area of monitoring and evaluation of the roles of HMOs and other players.

No matter the form of investigation, it is important to know that the level satisfaction should be seen as either acceptable or not. Government represented by NHIS therefore needs to ensure that the system is running properly. Satisfaction in health insurance scheme can only be judged to be satisfactory if the stakeholders particularly the beneficiaries (the ultimate consumers) consider it so. HMOs should understand that getting stakeholders aware and interested in their roles are major responsibility they should take.

This study also shows although social h health insurance was seen as been an important health financing mechanism, the roles and responsibilities of HMOs need to be strengthened beyond the current state so that those issues that bother on accessibility, affordability and equitable distribution of health needs across socioeconomic groups could be addressed and improved. Access requires that people who are enrolled should have health providers that are accessible to them when they need care. This can be related with Ghana’s experience where average individuals enrolled in their insurance scheme are significantly more likely to obtain prescription, visit clinics and seek formal health care when sick [31, 32]. HMOs should provide the summary operating guidelines to the beneficiaries so as to ensure that accessible health providers are chosen even at the process of registration. There is need to ensure that all basic health services are covered in the benefit package of the insurance scheme so that people in low socioeconomic groups that have health insurance coverage do not incur catastrophic costs when they utilize healthcare services.

### Study limitations

The study limitation was the fact that not many people were interviewed in the qualitative aspect of the study, especially from the government and NHIS. Categorization of the respondents was also difficult as many of them fall into small groups that were not options in the study instrument which made a good number of respondents to be grouped as others. Also, the study focused on just one state in one geographic area of the country. We hope that future studies on the same subject will be able to increase the scope for data collection.

## Conclusion

This investigation noted the roles but observed that there exists some level of inefficiency in the system. The reason for involving HMOs in the National Health Insurance Scheme was for them to reduce the excesses of the social system which does not drive efficiency. But the current situation of things indicates that the scheme still suffers inefficiency, which supports the evidence from other African countries that general health systems are too weak to efficiently and equitably deliver required interventions to people that need them [33]. Efforts that will aim at improving equity for health in Africa should therefore include methods that will ensure a more accessible and affordable health care for people irrespective of their socioeconomic status.

The quantitative and qualitative responses in the study have points of convergence. For instance, they both agreed that there is low satisfaction with the roles of HMOs. However, response of HMO managers in the qualitative arm did not support the argument. The HMOs generally believe that their roles are satisfactory given that they are providing private sector touch to the scheme. The study showed that HMOs are not very effective and have not been able to meet their objectives and again have not discouraged unwarranted practices by the providers in the scheme. It is necessary to note that some of the HMOs interviewed agreed that their roles are below expectation and would need to improve. Improving on the roles of HMOs in the insurance scheme should require introduction of performance based financing that enables the public sector remunerate the HMOs based on what they are able to contribute positively to the system [34].

The study makes it imperative for government to know that health insurance could be scaled up to other sectors of the economy, not just the federal employees. The result tells HMOs and indeed NHIS that more is desired from them for an improved system. Now that countries are striving towards UHC, Nigeria cannot afford to be left behind. This study is important in that it encourages the system to consider seriously the establishment of health insurance at the state and local government levels.

## Data Availability

Not applicable.

## References

[1] National Health Insurance Scheme Decree No 35. Law of the Federation of Nigeria: Error! Hyperlink reference not valid.

[2] Health Maintenance Organization Act. Ministry of Justice, USA. 1973. https://study.com/academy/lesson/health-maintenance-organization-act-history-summary.html.

[3] National Health Insurance Guidelines (2005): https//www/oseroghoassociates.com>articles> 105-national-health-insurance.

[4] Nishtar S (2004). Public-private ‘partnerships’ in health – a global call to action. Health Research Policy and Systems.

[5] Njau RJA, Mosha FW, De Savigny D. Case studies in public-private partnership in health with the focus of enhancing the accessibility of health interventions. Tanzan J Health Res. 2009;11(4):235–49. 10.4314/thrb.v11i4.50196 20734704

[6] Watson S (2010). Michael and Schoonmaker Michele.

[7] Liran E, Amy F (2017). Moral Hazard in Health Insurance: What We Know and How We Know It. NBER Working Paper No. 24055.

[8] Tynkkynen LK. Towards partnership?Studies on public-private collaboration in health care and elderly care services in Finland. Tampere: Tampere University Press; 2013.

[9] Tynkkynen L, Keskimaki I, Lehto J (2013). Purchaser-provider splits in health care – the case of Finland. Health Policy.

[10] NHIS, About us (2013). National Health Insurance Scheme.

[11] Baruwa E. Health financing functions: risk pooling. Cambridge: Health Finance and Governance, Abt Associates Inc, USAID; 2015.

[12] O’Donnell O, Van Doorslaer E, Rannan-Eliya R, Somanathan A, Adhikari S, Akkazieva B (2005). “Who pays for health Care in Asia?” EQUITAP working Paper1, Erasmus University, Rotterdam and Institute for Policy Studies, Colombo.

[13] Preker AS, Langenbrunner J (2005). Spending Wisely: Buying Health Services for the Poor.

[14] Tait A (2001). Mobilization of domestic resources for health through taxation: a sum-mary survey. Background paper 14 for working group three of the commission on macroeconomics and health.

[15] Kutzin J (2013). Health financing for universal coverage and health system performance: concepts and implications for policy. Bull World Health Organ.

[16] Emily G-w, Onno S. Achieving universal health coverage in Nigeria one state at a time: a public-private partnership community-based health insurance model. Washington DC: Brooke Shearer Working Paper Series; 2013.

[17] Mathauer I, Dale E, Jowett M, Kutzin J (2019). Purchasing health services for universal health coverage: how to make it more strategic?.

[18] Chima OA, Kara H, Johanna H. Towards universal coverage: a policy analysis of the development of the National Health Insurance Scheme in Nigeria. Health Policy Plan. 2014;9:1105–17. 10.1093/heapol/czu116 25339634

[19] Leech NL, Onwuegbuzie AJ (2009). A typology of mixed methods research designs. Qual Quant.

[20] Wao Hesborn O, Onwuegbuzie Anthony J (2011). A mixed research investigation of factors related to time to the doctorate in education. Int J Doctoral Studies.

[21] Creswell JW (2007). Qualitative inquiry and research design: choosing among five approaches.

[22] National Population Commission Nigeria (2014). National Bureau of statistics (web). Enugu State Nigeria.

[23] Cochran WG. Sampling Techniques. Aulf. New York: Willey; 1963.

[24] Toyin A. Repositioning Health Insurance in Nigeria: Prospects and Challenges. Int J Health Sci. 2014;2(2):151–62.

[25] World Bank (2018). Universal Health Coverage Overview – World Bank Group.

[26] Fenny AP, Yates R, Thompson R (2018). Social health insurance schemes in Africa leave out the poor. Int Health.

[27] Spaan E, Mathijssen J, Tromp N (2012). The impact of health insurance in Africa and Asia: a systematic review. Bull World Health Org.

[28] Carapinha JL, Ross-Degnan D, Desta AT, Wagner AK (2011). Health insurance systems in five sub-Saharan African countries: medicine benefits and data for decision making. Health Policy.

[29] Federal Ministry of Health (2005). Nigeria Health Financing System Assessment, World Bank.

[30] Kutzin J, Yip W, Cashin C. Alternative financing strategies for universal health coverage. Handb Glob Health Econ Public Policy. 2017;1:1–43. https://books.google.fr/books?hl=fr&lr=&id=l3rQDAAAQBAJ&oi=fnd&pg=PT293.

[31] Blanchet NJ, Fink G, Osei-Akoto I (2012). The effect of Ghana’s National Health Insurance Scheme on health care utilisation. Ghana Med Journal.

[32] Kusi A, Enemark U, Hansen KS (2015). Refusal to enroll in Ghana’s National Health Insurance Scheme: is affordability the problem?. Int J Equity Health.

[33] Kirigia Joses, Barry Saidou (2008). Health challenges in Africa and the way forward. International Archives of Medicine.

[34] Ridde V, Gautier L, Turcotte-Tremblay AM, Sieleunou I, Paul E (2018). Performance-based financing in Africa: time to test measures for equity. Int J Health Serv.

